# Neurolysis for Treatment of Infraorbital Neuropathy

**DOI:** 10.1155/2017/2389354

**Published:** 2017-11-14

**Authors:** Ahmet Mahli, Demet Coskun

**Affiliations:** Department of Anaesthesiology and Reanimation, Gazi University Faculty of Medicine, Ankara, Turkey

## Abstract

Two patients, a woman aged 34 and a man aged 56, were included in the study. They reported the existence of pain in the areas of the infraorbital nerve, which, over the last four to five years, aggravated by the stimuli of eating, laughing, and being touched. 2 ml of 0.5% lidocaine was administered to these patients six times percutaneously. It was observed that the ease of pain lasted until the local anesthesia lost its effect, and the pain resumed its original intensity. Neurolysis with 0.5 ml of 50% ethanol was applied to the infraorbital nerve. This procedure was applied to the first patient twice and three times to the second. Thereafter, the patients were cured completely. For the treatment of idiopathic chronic infraorbital neuropathy, the neurolysis of the infraorbital nerve using 50% ethanol could be considered as an effective treatment alternative.

## 1. Introduction

Infraorbital nerve neuralgia is an uncommon element for facial pain. It is essential to manage facial pain appropriately as it often causes pain, and the stimuli of eating, laughing, and being touched result in irritation. Among a number of management options present for facial pain, intervention at the branches of the trigeminal nerve has been proved to be clinically useful [1, 2]. Blocking and then alcohol neurolysis of the infraorbital nerve using a closed method has never been discussed. Accordingly, for the treatment of facial pain, we agreed to assess the safety and efficiency of alcohol neurolysis of the infraorbital nerve. To this aim, we used a closed method in two patients suffering from idiopathic chronic infraorbital neuropathy.

### 1.1. Case Report 1

A woman aged 34 complained about brief stabbing pain in the innervated right areas of the infraorbital nerve that lasted for four years. The pain was aggravated by the stimuli of eating, laughing, and being touched. The intensity of the pain was 5 out of 10 on the visual analog scale (VAS, with 0 indicating no pain and 10 the worst pain imaginable). She also suffered from infrequent exacerbations which lasted about an hour and the pain intensity of which was 9/10 on the VAS.

### 1.2. Case Report 2

A man aged 56 was included in the study. He complained about brief stabbing pain in the innervated right areas of the infraorbital nerve that had lasted for five years, and the pain was defined to be aggravated by the stimuli of eating, laughing, and being touched. The intensity of the pain was 5 out of 10 on the visual analog scale (VAS, with 0 indicating no pain and 10 the worst pain imaginable). He also suffered from infrequent exacerbations which lasted about half an hour and the pain intensity of which was 9/10 on the VAS.

Before consulting our pain service, both patients had also been assessed by the departments of neurology, otorhinolaryngology, and maxillofacial surgery. The patients had first consulted to the department of neurology, where they were clinically examined and their diagnostic tests were performed. Magnetic resonance imaging and facial computed tomography scans had also been performed, yielding no pathological findings. Both of them stated that they had never previously experienced any trauma and undergone any face or tooth operation. Then, consultation was asked from the departments of otorhinolaryngology and maxillofacial surgery. These departments were also not able to identify any pathology. Thereupon, the neurology clinic diagnosed these patients with idiopathic infraorbital neuropathy and pharmacological treatment was started, from which neither patient could benefit.

When both these patients presented to our pain clinic, the case was as follows: the patients reported that they could not get rid of their pain although they received carbamazepine, diphenylhydantoin, and baclofen therapy. They also stated that it was confirmed by a dentist that they did not have any dental problems. The neurological examination of both patients revealed that the pain could be caused by the palpation of the nerve in the infraorbital notch. During the examination, no other concomitant data of interest were observed. It was concluded that, as the pains were in the region innervated by the infraorbital nerve, the patients had idiopathic infraorbital neuropathy. Consequently, by taking the condition of the patients into consideration, we had the same diagnosis with the neurology clinic. However, the patients had not benefited from the pharmacological treatment, which was the first option in such cases. Therefore, based on our previous experience with such patients [3], we decided to perform alcohol neurolysis of the infraorbital nerve on the patients for the treatment of facial pain.

We administered 2 ml of 0.5% lidocaine hydrochloride over six times percutaneously to these patients. It was seen that the patients were free from pain until the local anesthesia lost its efficiency, but then the pain resumed at its usual intensity. Thus, in order to ease the pain definitely, the patients were recommended the application of neurolysis and informed on the side effects and complications that might occur. Then, upon the receipt of confirmations of the patients, application of neurolysis was started. After identifying the location of infraorbital foramen on the patient's face using thumb nail, a 22-gauge needle was forwarded down to the point where it touched the periost. In the infraorbital foramen, nerve, artery, and vein are found together. In order not to destroy the perfusion of this area, the needle was withdrawn 1 mm immediately after it touched the periost. After the negative result of the aspiration test, 2 ml of 0.5% lidocaine was injected. The needle was held up still for a whole minute until when the patient confirmed that the pain was gone, and then 0.5 ml of 50% ethanol was injected.

For the first patient, the same procedure was repeated 8 months after the first time, when the patient suffered from mild pain. For the second patient, however, the same procedure was repeated twice: one after six months and the other after a year as he still suffered from moderate pain. During the patients' clinical follow-up, they were scheduled for an evaluation once in six months for the following two years. Besides, they were advised to call in immediately in the event of any pain. At the end of the clinical follow-up, these two patients reported that they did not experience such pain any longer.

## 2. Discussion

By virtue of its ophthalmic, maxillary, and mandibular divisions, the trigeminal nerve supplies the face and anterior portions of the scalp. The maxillary nerve is wholly sensory. The maxillary nerve gives seven branches within the pterygopalatine fossa, and it passes through the infraorbital canal as it continues. It exits onto the front of the maxilla through the infraorbital foramen. At this very point, the maxillary division is now known as the infraorbital nerve. The terminal branches of the infraorbital nerves on the face are the inferior palpebral, lateral nasal, and superior labial branches [4, 5].

When infraorbital neuralgia is in question, other symptomatic causes should be eliminated to be able to assign a specific diagnosis. The presence or absence of any history of any traumatic episode should be determined. If such a history is not in question, other secondary causes, mainly neoplasms that can cause these symptoms due to haematogenous, lymphatic, or perineural spread, should be ruled out. Therefore, imaging studies are essential for all these patients. Upon the elimination of all the above-mentioned possible causes, primary neuralgia can be considered [5, 6].

Medical treatment for infraorbital neuralgia generally includes analgesic, anti-inflammatory, anti-epileptic, or antidepressant drugs; however, it may be unmanageable by medical treatment [2]. Among other alternatives for therapy, there is electrical transdermal nerve stimulation for extremely resistant cases [4]. All these treatment alternatives provide symptomatic and temporary recovery. It has been reported that persistent hyperesthesia due to trauma-induced infraorbital nerve compression cases were successfully treated by means of surgical decompression operations, despite a possible need for a second operation. However, we are of the opinion that this treatment method is an invasive one [7–9].

Cases where terminal branch neuralgia of the trigeminal nerve is in question are quite rare, and they frequently appear in the form of continuous pain. However, cases where the trigeminal nerve is centrally involved are generally connected to painful paroxysms [10]. Considering these neuralgias and palpating the nerve territories corresponding to the painful area may provide effective treatment alternatives for symptoms that are frequently persistent and incapacitating. One of the essential diagnostic criteria for such neuralgias is the nerve block, and therefore, it may be suggested as a first therapeutic step. Thus, we performed infraorbital nerve block 6 times on each patient for treatment and once just before neurolysis for diagnostic purposes.

When the presence of peripheral nerve injury is obvious, a direct treatment on this area would be appropriate. If trauma or amputation of a body part is in question, the cut ends of the neurons search desperately for the nerve trunk which is missing, and in the end, they may curl around themselves in a whirl-like fashion or become nested in either a scar or some other soft tissues. These bulbous collections of nonmyelinated neurons are called neuromas. Neuromas do not act like normal sensory receptors. They often cause an excessive response with sharp lancinating pain along the affected nerve when they are stimulated. In the meantime, both mechanosensitivity and thermosensitivity may be observed [3, 10].

In the treatment of pain secondary to peripheral nerve lesions, many treatment methods have been reported to be useful. Despite the fact that it is far from being a routine procedure, the neurolytic block (neurolysis) has been reported to provide patients with secure and effective relief for pain that does not respond to conventional treatments. In order to achieve neurolysis, the neurolytic agent is injected to intentionally destroy a nerve or nerves and thus interrupt nociceptive pathways for weeks or months. The use of different agents for chemical neurolysis is suggested in literature. Alcohol is one of the agents used in this procedure [3].

Neurolysis is a medical surgery method performed by the injection of a neurolytic agent on the nerve. It other words, it is the chemical destruction of the nerve without incision in order to interrupt the transmission of nerve signals. The side effects or complications depend on the nerve on which neurolysis is performed. Therefore, prior to neurolysis, whether the nerve in question is a motor nerve or a sensorial nerve or both should be known, and the formations it innerves should be well understood. The procedure is performed according to the topographical anatomy of the nerve that will be neurolysed. Before neurolysis, the patient should be informed regarding the senses that will disappear, and the functions that will be lost following neurolysis and consent should be received.

In these cases, the infraorbital nerve on which we performed neurolysis was one of the sensorial branches of the maxillary branch of trigeminal nerve, which performs innervation following exit from the infraorbital canal. The infraorbital nerve is found within the canal with vein and artery. When neurolysing this nerve, entry into the infraorbital canal should strictly be avoided. Thus, the complications that may occur will be prevented. The patient should be informed that following the neurolysis procedure, there will be numbness or tingling at the region innervated by the nerve.

Both these patients who applied to our pain clinic suffering from facial pain had previously been given proper standard pharmacological treatment; yet neither of them benefited from it even though the time passed was sufficient for such treatment. Therefore, based on our previous experience, we decided to perform alcohol neurolysis of the infraorbital nerve procedure on these patients. Prior to the decision for the performance of neurolysis procedure on the patient, it is necessary to make sure that all steps regarding standard pain treatment are taken and yet the patient does not benefit from any of those steps. It is recommended that for the treatment of head and face neuralgia, the conventional treatment involves carbamazepine, diphenylhydantoin, or baclofen. However, it is stated that if medical measures fail, radiofrequency treatment of the ganglion or microsurgical decompression of the trigeminal root is appropriate [11]. For pain treatment in such neuralgia patients, the method of treatment involving peripheral neurolysis with alcohol is used progressively less. Nevertheless, in the cases we present, it can be considered that when compared with radiofrequency treatment method, alcohol neurolysis procedure is less harmful and therefore a superior treatment option as it does not destroy the vein and artery structure within the infraorbital canal. Once the infraorbital nerve exits the canal, it gives off side branches to the lower eyelid, cheek, and lip regions on that part. We apply the neurolysis method to the side branches that occur after the infraorbital nerve exits the canal. However, if radiofrequency method is used before entering the infraorbital canal, the chances of catching these side branches selectively decrease. For this reason, if the nerve in question exhibits several branches, which happens to be the case in our study, we are of the opinion that preferring neurolysis method over radiofrequency method would be more convenient.

To our knowledge, our study is the first one to report on neurolysis of the infraorbital nerve by using a closed method. For our patients, in order to cure infraorbital neuropathy, we administered 2 ml of 0.5% lidocaine six times over three-day intervals (therapeutic local anesthesia). However, it was observed that after a few hours, the patients' pain resumed to its initial intensity. Therefore, for the treatment of the pain for good, we decided to perform neurolysis using alcohol. The patients were given information on the procedure to be made, and then, 0.5 ml of 50% ethanol neurolysis was applied to the infraorbital nerve. We repeated the procedure for a second time for the first patient, and for the second patient, we had to repeat the procedure two more times. Then, the pain went for good.

In cases where conservative methods do not work to eliminate pain within six months, and if a damage occurring on the infraorbital nerve is in question, we recommend the application of neurolysis to the infraorbital nerve. For the treatment of idiopathic chronic infraorbital neuropathy, the neurolysis of the infraorbital nerve using 50% ethanol could be considered as an effective and safe treatment.
